# Multi-Level Cross Residual Network for Lung Nodule Classification

**DOI:** 10.3390/s20102837

**Published:** 2020-05-16

**Authors:** Juan Lyu, Xiaojun Bi, Sai Ho Ling

**Affiliations:** 1 College of Information and Communication Engineering, Harbin Engineering University, Harbin 150001, China; lvjuan@hrbeu.edu.cn (J.L.); Bixiaojun@hrbeu.edu.cn (X.B.); 2 College of Information Engineering, Minzu University of China, Beijing 100081, China; 3 School of Biomedical Engineering, University of Technology Sydney, Ultimo, NSW 2007, Australia

**Keywords:** lung nodule classification, computed tomography, residual convolutional neural network, ternary, binary

## Abstract

Computer-aided algorithm plays an important role in disease diagnosis through medical images. As one of the major cancers, lung cancer is commonly detected by computer tomography. To increase the survival rate of lung cancer patients, an early-stage diagnosis is necessary. In this paper, we propose a new structure, multi-level cross residual convolutional neural network (ML-xResNet), to classify the different types of lung nodule malignancies. ML-xResNet is constructed by three-level parallel ResNets with different convolution kernel sizes to extract multi-scale features of the inputs. Moreover, the residuals are connected not only with the current level but also with other levels in a crossover manner. To illustrate the performance of ML-xResNet, we apply the model to process ternary classification (benign, indeterminate, and malignant lung nodules) and binary classification (benign and malignant lung nodules) of lung nodules, respectively. Based on the experiment results, the proposed ML-xResNet achieves the best results of 85.88% accuracy for ternary classification and 92.19% accuracy for binary classification, without any additional handcrafted preprocessing algorithm.

## 1. Introduction

In 2018, more than 1.7 million people died from lung cancer, which accounted for the highest proportion of deaths of all kinds of diseases, reaching 18.4%. Meanwhile, lung cancer led to the highest number of new cases in 2018, at more than 2 million cases [1]. Lung cancer has the highest death rate (26%) among the top four major cancers with a low five-year survival rate of 18%, according to the report from American Cancer Society in 2019 [2]. The reason for the low survival rate is that the clinical symptoms of lung cancer usually present in the advanced stage [3]. Hence, the early diagnosis is essential, which can improve the five-year survival rate to above 90% and also enhance the chance of cure.

**Figure 1:**
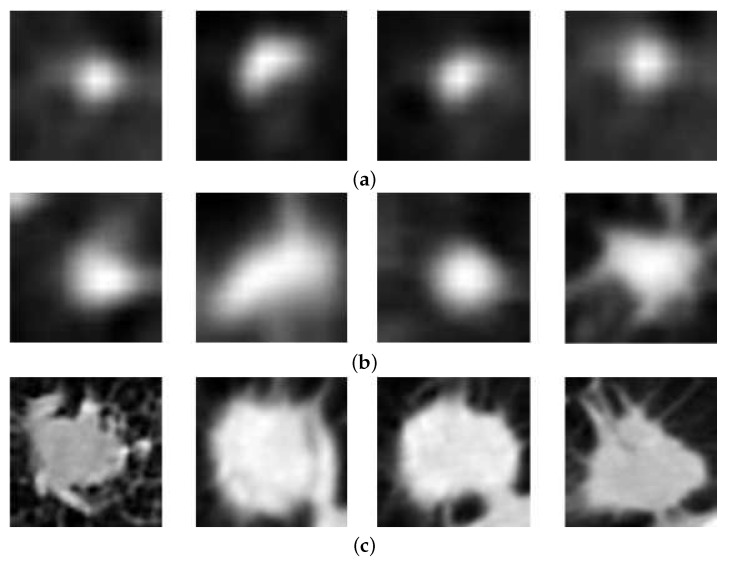
Samples for three classes: (**a**) benign nodules; (**b**) indeterminate nodules; and (**c**) malignant nodules.

A lung nodule is an abnormal growth on the lung that is smaller than 30 mm [4]. If it is larger than 30 mm, it is called lung mass and has a higher chance of being cancerous [5]. Lung nodules can be benign and malignant. Benign nodules are noncancerous, and generally have more regular shapes, smoother contours, smaller size, and fatter tissue density than cancerous nodules [4], as shown in Figure 1a. Benign nodules are usually asymptomatic and do not have the risk of spreading to other body parts. Malignant nodules are cancerous, and have more irregular morphologies, higher density, and are larger than benign nodules and show the evidence of nodule spiculation [4], as shown in Figure 1c. Malignant nodules can spread to other organs or tissues and proliferate quickly, consequently causing physical discomfort and even threatening life. There are also some nodules that are hard to be consistently diagnosed among radiologists who have different experiences, as shown in Figure 1b. They may have similar morphologies as both benign and malignant nodules. For example, patients may have solitary or single pulmonary nodule as well as multiple nodules. Therefore, doctors can provide accurate prognosis and treatment for patients through accurate diagnosis of each stage of the nodules.

The diagnosing of lung cancer is assisted in computer tomography (CT). The physicians give interpretations and inferences relying on their experiences. However, inescapably, they are affected by subjectivity, or even fatigue when they read many CT images in a day [6]. At present, a computer-aided diagnosis (CAD) system has become an important measure and assistance to mitigate their work, especially the deep learning methods [7]. Deep learning is popular and performs impressively on numerous aspects of diagnostic modes, such as medical imaging [8], EEG, ECG [8], etc. Due to the extensive and in-depth development of deep learning in computer vision, the progress of medical imaging benefits from this tend. There are plenty of techniques used in medical image processing, such as convolutional neural network (CNN) [9], transfer learning [8], attention mechanism [10,11], generative adversarial networks (GAN) [12], unsupervised learning [13], etc. for disease classification, detection, segmentation, reconstruction, and so forth [14,15].

Likewise, lung cancer diagnosis and classification has acquired some achievements from CNNs [14]. For binary classification, Shen et al. [16] proposed a multi-scale CNN (MCNN) to train three scales of input images. These images are inputted into a weight shared network which can be regarded as one CNN network, and the network is a standard shallow CNN. Therefore, the classification performance is limited. Then, to improve the classification performance, they proposed a new structure named multi-crop CNN (MC-CNN) [17]. This structure is mainly based on the multi-crop pooling method, which crops the center of the feature maps twice to quarter size. Next, it uses different times of max-pooling to produce multi-crop features. However, multiple downsampling causes the loss of useful features. They achieved 87.14% for binary classification. Nóbrega et al. [18] studied the performance of transfer learning for lung nodule classification based on 11 kinds of existing and widely used models, such as VGG, ResNet, DenseNet, and NASNElarge. Then, they classified the extracted deep features using six categories classifiers, e.g. Support Vector Machine (SVM), Random Forest (RF), etc. Finally, they obtained the best accuracy of 88.41% and AUC of 93.19% by ResNet50 with SVM-RBF. Dey et al. [19] proposed four two-pathway networks based on the 3D CNN: basic 3D CNN, 3D multi-output CNN, 3D DenseNet and 3D multi-output DenseNet (MoDenseNet). All the networks are pre-trained on the ImageNet. The two-pathway networks are trained by the two-view of inputs. Finally, they gained the best accuracy of 90.47% by the 3D multi-output DenseNet. However, they only applied 686 nodule samples for both training and testing processes.

In 2019, a deep local–global network was proposed for lung nodule classification [20]. In this structure, the residual blocks and the non-local blocks are connected alternately, followed by a global average pooling layer and sigmoid classifier. However, they did not change the size of the features during the entire process, which is all through the same as inputs. Then, suddenly the size is changed to 1 × 1 using global average pooling. In this way, there are many redundant features during the convolutions and information is lost when using the global average pooling, which is not suitable for extracting useful features for the classification. El-Regaily et al. [21] proposed a multi-view CNN to reduce the false positive rate for lung nodule binary classification. They obtained three views from the nodule’s 3D model: axial, coronal, and sagittal. Then, these three views are inputted into three complete CNNs, training their softmax classifiers individually. Finally, they fused three outputs to get the final classification result using a logical OR operation. However, the CNNs they used are three layers of the standard network. Even though they used a logical OR operation to select the best classifier, the classification ability of each classifier is limited. Hussein et al. [22] proposed to classify the lung and pancreatic tumors using both supervised and unsupervised methods. For the supervised model, they introduced a multi-task learning method based on a transfer-learning-based 3D CNN, which combines the classification and attribute score estimation. They obtained an accuracy of 81.73% for binary classification. For the unsupervised learning, they used the proportion-support vector machine to get the initial labels and label proportions. Then, they classified the tumors based on them. They achieved a 78.06% accuracy for lung nodule classification. However, the dataset they used only includes 1144 nodules. Liu et al. [23] proposed a multi-task network for lung nodule binary classification and attribute score regression to enhance the performance of two tasks. Then, they applied a Siamese network with a margin loss to learning to rank the most malignancy-related features to improve the distinguishing ability of the network. They achieved an accuracy of 93.5%, a sensitivity of 93.0%, a specificity of 89.4%, and an AUC of 97.90%. However, they only used 1250 images. Although they used five-fold cross-validation to evaluate the effectiveness and robustness of the network, the quantity of the dataset is only 5.7% of ours. Small datasets may cause a lack of universality.

However, not all nodules have distinguishing characteristics to identify. There are some indeterminate nodules for experienced professionals. They need to combine other measures to make a definite diagnosis. Therefore, identifying these nodules is also essential to the treatment and cure of the patients. In this task, we label this class of nodules as indeterminate for ternary classification. Shen et al. [17] also considered the ternary classification, and the accuracy is only 62.46%. In our previous work [24], we proposed a multi-level convolutional neural network (ML-CNN), which consists of three levels two-layer CNNs, and achieved an accuracy of 84.81%.

As lung nodules have different sizes and various morphologies, inspired by Inception [25], multi-scale features of input are gaining attention. While different from Inception, we designed a multi parallel levels structure based on the ResNets [26], and all levels are with different scales of convolutional kernels. Moreover, to share the information among all the levels, we propose to connect the residuals cross levels additionally. Overall, in this paper, we propose a multi-level cross ResNet (ML-xResNet) to enhance the performance of lung nodule malignancy classification in thoracic CT images, including ternary and binary classifications. We classify the nodules into benign, indeterminate, and malignant in ternary classification, and then we delete indeterminate nodules to get the binary dataset. Our approach was evaluated on the Lung Image Database Consortium and Image Database Resource Initiative (LIDC-IDRI) database [27].

ML-xResNet is based on the following ideas. Since the lung nodules have various sizes and morphologies, using a fixed size of filter to extract their features causes information loss or insufficiency. To solve this problem, we design a novel structure named multi-level cross residual (ML-xRes) block. The ML-xRes block consists of three parallel residual blocks in the same structure but different convolution kernel sizes for extracting multi-scale features of the input nodules. Moreover, due to the lack of fusion among all scales of features, to improve the efficiency of extracting multi-scale features of the inputs, we propose to connect the residuals not only in the same levels but also cross other levels. In this way, with the training process, the information on all levels is shared and combined. Then, we insert the ML-xRes blocks into our multi-level structures. Except for the blocks, others are the typical convolution structures with max-pooling layers. Finally, we fuse the outputs of three levels using the concatenate technique and classify the nodules using a softmax classifier.

The rest of the paper is organized as follows. Section 2 describes the details of the proposed ML-xResNet. Section 3 introduces the materials we used. Section 4 and Section 5 show the experiment results and discussion. Section 6 concludes the paper.

## 2. Methods

In this section, we describe the main components of the proposed ML-xResNet, the ML-xRes block. Then, we introduce the proposed multi-level cross residual neural network in detail.

### 2.1. Multi-Level Cross Residual Block

Lung nodules with different malignancy are on different scales and various morphologies. For small size nodules, if using a large filter to extract their features, after several times of down sampling, the feature maps only contain few pixels of the small object, while the majority is irrelevant information or even blank. It leads to loss of details and low classification accuracy for small nodules. Meanwhile, for large size nodules, if extracting features using a small filter, even after several times of down sampling, the receptive fields are too small to capture the holistic information of the nodules, which also causess a low classification accuracy. Therefore, we need to use a filter with a small receptive field for small size nodules, and a large filter for large size nodules. However, the input nodule sizes are random and unpredictable, and it is impractical to design a particular filter size for each scale of objects.

We propose to address this problem in three aspects. Firstly, we design a multi-level structure to extract multi-scale features of the nodules. For input, using different sizes of filters can extract its different scales of features. The multi-level structure is constructed by parallel convolutions with small and large kernels; thereby, each level extracts a scale of features. Thus, we have more appropriate reception fields for both small and large nodules than a single scale filter. Then, for the convolution, we adopt the residual technique. The excellent performance of ResNet has been proved in both computer vision and medical imaging tasks, which can efficiently solve the problem of gradient vanishing. By connecting the residuals from former layers, it can also help to offset the loss of details for small nodules or to gain more holistic information for large nodules. However, the different scales of features in those levels are independent. To further enhance the details or the holistic information of the nodules, we propose to fuse the multi-scale features. It is proved that the fusion of different scales of features is helpful to identity multiple scales of objects [26,28,29]. Therefore, we combine all scales of features through residuals connections. To share information between different levels, we propose to connect the residual information not only at the same level but also across other levels. In this way, with the process of training, the different scales of features are fused adequately.

**Figure 2:**
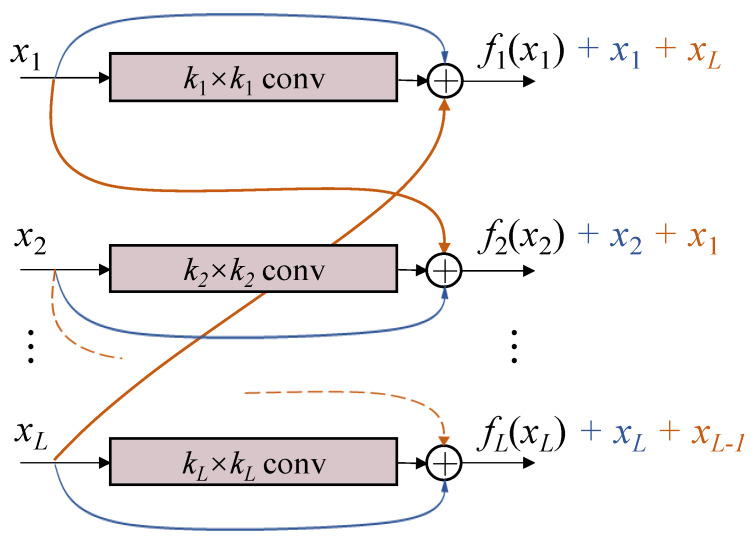
The illustration of the multi-level cross residual block. The residuals ***x*** are not only connected to the output of their existing layers, but also added to the cross layers. The residual in the last layer is connected to the output of the first layer. The conv means convolution + BN + ReLu.

The proposed block is shown in Figure 2, named multi-level cross residual (ML-xRes) block. Considering the computational cost, instead of connecting a residual with all the other levels, we only connect it with its next level or with the first level if the residual is in the last level. Therefore, the final outputs of the ML-xRes block are defined as

(1) $$\left\{\begin{array}{c} o_{1}=f_{1}\left(x_{1}\right)+x_{1}+x_{L},i=1\\ o_{i}=f_{i}\left(x_{i}\right)+x_{i}+x_{i-1},i\in \{2,3,\ldots ,L\} \end{array}\right.$$

where we assume that there are total *L* parallel levels in the block, the inputs of this block are $x_{1}$, $x_{2}$, …, $x_{L}$. Their corresponding outputs are defined as $f_{1}\left(x_{1}\right)$, $f_{2}\left(x_{2}\right)$, …, $f_{L}\left(x_{L}\right)$, respectively. The *i* means the *i*th level and $o_{i}$ means the output of the *i*th level. The convolution kernel size of each level is $k_{i}$. Based on the applications, readers can design their own convolution kernel size of each level and the number of levels.

### 2.2. Multi-Level Cross Residual Neural Network

**Figure 3:**
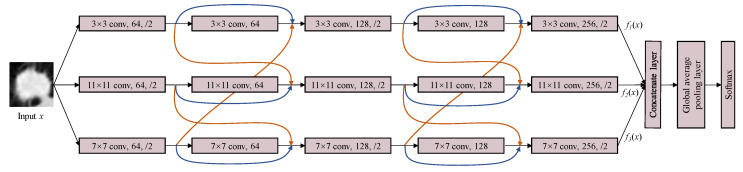
The diagram of the multi-level cross residual neural network. The input x is inputted into three parallel levels which have the same structure but different convolution kernel sizes. ML-xResNet contains two xRes blocks and three normal convolutional layers as well as max-pooling layers. Then, fusing the outputs of three levels by the concatenate layer, and through a global average pooling layer, the final result can be obtained by the softmax classifier.

Based on the proposed ML-xRes block, we design a multi-level cross ResNet (ML-xResNet) to classify the lung nodule malignancy. The architecture of the three-level-xResNet is shown in Figure 3. The ML-xResNet consists of three parallel levels of five-layer ResNets, including two ML-xRes blocks. The number of levels of structure and layers was determined by trial and error through experiment, while we also analyzed the causes. The residuals in the ML-xRes block are connected only with the adjacent levels; after the first block, the output of each level is composed of three elements, namely the output and input of the current layer and the input of adjacent layer. These three features are only from two of the three levels. Then, through the second block, all the levels’ features in different scales are fused completely. Two blocks are the most suitable for our task based on the experiment result. The convolution kernel size of each level is 3×3, 11×11, and 7×7, respectively, for extracting multi-scale features of the input. For classification tasks, 3×3 is the most common convolution kernel size. However, some of the lung nodules in this task occupy huge areas of the images. Therefore, we need to design lager filters for those nodules. Nonetheless, if using the large filter size, the computation would be increased. To balance the performance and efficiency of the network, we propose to use the above-mentioned three scales of filters as the size of the images in our task is 52×52. For the odd layers, a dropout layer and a max-pooling layer follow the convolution layer. The dropout keep rate is set at 0.8, as the experiment also showed that it is the most reliable number in our task. The output of an even layer as well as the residuals from the same level and adjacent level are of the same dimensions and are connected by elementwise addition. The two ML-xRes blocks can share information between three levels to improve the identifying ability of the features. Then, we fuse all the features at the end of three levels using concatenation. The outputs of the fifth layers of three levels are concatenated together, and then, we use a global average pooling layer to summarize the information. At last, a softmax classifier is used to identify the nodules’ malignancies.

Since we have smaller data size, we use a smaller network to extract features than the traditional ResNet, but it is efficient for our task. The classifier identifies different classes objects based on both semantic and detail features. Small filters usually can develop more detail information of the input but are lacking the ability of the semantic representation. For the large filters, it is the opposite. The proposed ML-xResNet tackles this problem through the multi-scale feature extracting and fusion techniques. After the fusion of all scales of features, the details and the semantic information of a nodule can remedy the characteristics of each other, thus improving the classification performance. Moreover, when we design the filter sizes, to alleviate the overfitting problem, we do not employ too large filters. As we know, the overfitting problem reduces the classification accuracy. In the meantime, compared to the standard convolution, the residual technique of the ResNet can also improve the classification performance. Overall, the proposed ML-xResNet can enhance the classification performance of the lung nodule malignancy.

## 3. Materials

In this section, we introduce the database we used and how we obtained our training and testing datasets. Then, we present the experimental setup of the experimental environment, the setting of the hyperparameters, and the assessment criteria.

### 3.1. Data

The database we used in this work is the publicly available Lung Image Database Consortium and Image Database Resource Initiative (LIDC-IDRI) [27,30]. In this database, 1010 patients participated, and in total 1018 cases are presented. All the cases were scanned in thoracic CT and comply with the standard of the Digital Imaging and Communications in Medicine (DICOM). There are three kinds of nodules: nodule ≥ 3 mm, nodule < 3 mm (small nodule), and non-nodule ≥ 3 mm. The data were annotated by four radiologists independently. The images in this database have a uniform in-plane pixel size of 512 × 512.

### 3.2. Data Setup

According to the annotation documents, only nodules larger than 3 mm have malignancy annotations, thus we excluded 142 cases with an only small nodule or non-nodule markings as noted in the nodule collection report [31]. Besides, there are 11 cases we deleted as some information is missing. Finally, the proposed methodology was applied to 865 CT scans. As annotations in the database, there are five different ratings of the malignancy levels ranging from 1 to 5: Levels 1 and 2 are considered as the benign nodule, Level 3 as the indeterminate nodule, and Levels 4 and 5 as the malignant nodule. As every nodule was annotated by more than one radiologist, the diagnosis was attributed to the class with the highest frequency. If two classes both have the highest frequency, we considered this nodule as an indeterminate nodule. Consequently, we gained 5376 benign, 11,000 indeterminate, and 22,499 malignant nodules from the database, respectively.

In this research, we set the ratio of the training and testing data as 85:15. We solved the unbalance between the nodule numbers in each case in the following way. We kept all the indeterminate nodules, doubled the number of benign nodules for the training set, and halved the number of malignant nodules randomly. For the benign nodules, we rotated the training data, and then added the original training benign nodules. Finally, we obtained 9139 benign nodules for training. Therefore, the final number of nodules for each class was 10,752, 11,000, and 11,249, respectively, for the ternary classification. Regarding the binary classification, we only deleted the indeterminate nodules to classify the benign and malignant nodules. All nodules were cropped based on the annotations and resized uniformly to 52 × 52 pixels since using the original size of the images is computationally expensive.

### 3.3. Experimental Setup

Our experiment was implemented in Python code using Tensorflow [32] and Tensorlayer [33] deep learning libraries, running on a machine with an Intel Core i7-8700K processor, 31.3 GB memory, GeForce GTX 1080 Ti 16 GB GPU, and Ubuntu 16.04 operating system.

The ML-xResNet was applied to both ternary and binary classifications. The corresponding parameters were set as followings. We trained the network in 300 epochs with a batch size of 200. The initial learning rate was 0.001, the momentum was 0.9, and the weight decay was $5\times 10^{-4}$. We took the cross-entropy as the loss function [34]. Moreover, the dropout parameters used in this network were all set as 0.8. For the ternary classification, we evaluated the performance of the proposed method with accuracy as well as the six-fold cross-validation. For binary classification, besides the accuracy, we also added sensitivity, specificity, and Area Under Curve (AUC) to evaluate the performance of our network, where the curve presents the Receiver Operating Characteristic (ROC) curve. They are defined as follows:

(2) Accuracy=(TP+TN)/(TP+FP+FN+TN)

(3) Sensitivity=TP/(TP+FN)

(4) Specificity=TN/(TN+FP)

where TP is true positive, FN is false negative, TN is true negative, and FP is false positive.

## 4. Results

We conducted several experiments to evaluate the performance of the proposed ML-xResNet. We carried out both ternary and binary classifications in our experiments.

### 4.1. Ternary Classification

We introduce how to select an efficient configuration from diverse structures and hyper parameters based on ML-xResNet. Thereafter, we compare our method with the state-of-the-art models to illustrate the significant improvement of ML-xResNet.

#### 4.1.1. The Exploration of ML-xResNet Structure

For the proposed multi-level parallel structure, we first evaluated the three-level xResNet we adopted by comparing it with different levels of networks. Except for the level numbers, all of these networks have the same structure and hyperparameters. To choose an optimum level of ML-xResNet, we compared the accuracies of 1–4-level structures. Since the kernel sizes we used in the three-level xResNet are 3 × 3, 11 × 11, and 7 × 7, the kernel sizes of compared single- and two-level network were one of or two of them, and we compared the results from all the permutation and combination. For the four-level structure, we took an additional level with kernel size of 5 × 5 along with the other three kernel sizes. The comparison results are shown in Table 1. We simply use S_S to present the combination of different kernel sizes. It is shown that, for the single-level networks, the model with a filter size of 11 obtains the best result, whereas the network with a filter size of 3 has the lowest performance. For the two-level architecture, the combination of 3_11 achieves the best result. As the number of levels increase from one to three, the classification accuracy increases. However, in contrast, when the number of levels is increased to four, the performance is not improved. Overall, the multi-level structures are all better than the single-level network, and our proposed three-level structure is the best for solving our ternary classification problem. The reason is, compared with single-level network, the multi-level structures can identify more characteristics of different kinds of nodules by extracting and fusing multi-scale features of the inputs. Moreover, compared with two levels model, the proposed ML-xResNet can combine more scales of features, which makes the network have better generalization. However, when there is an additional level, the adverse effect outdoes the improvement of one more scale of features, which causes a decrease of the accuracy. It indicates that the proposed ML-xResNet has the most appropriate number of levels.

Then, we compared ML-xResNet containing two ML-xRes blocks with the networks with different numbers of ML-xRes blocks. All networks have three-level xResNet. The comparison results are shown in Table 2. The connections of the ML-xRes blocks and standard convolution layers are alternated, and the first and last layers are standard convolution layers. Therefore, for clarity, we also show the numbers of features of each layer in Table 2. It shows that, with the number of the block increasing from one to two, the performance is enhanced with a more than 4% improvement. However, when the number of blocks continues to increase to four, the accuracy degresses more and more rapidly. The main reason is that the increasing of the blocks causes the increasing of the parameters of the network, and the problem of overfitting is raised. Further, the overfitting leads to a worse generalization of the networks. It reveals that our ML-xResNet containing two blocks has the best representation for all kinds of nodules. The reason is that the features extracted by our model have more details compared with the models with three and four blocks since they have more down-sampling layers, which lead to the loss of details of the nodules. Inversely, the network only with one ML-xRes block is down-sampled once. However, it does not have enough abstract features to present semantic information. Therefore, for this task, the proposed structure with two ML-xRes blocks has the best representation power.

Subsequently, since we adopted dropout layers following each odd convolution layers, to evaluate the performance, we compared it with the networks having different dropout keep rates as well as a network which contains dropout layers after all the convolutional layers. The comparison result is shown in Table 3. If we do not use dropout layer in our model, i.e. we use dropout layers which keep all the neurons, the classification accuracy is lower than our model using the keep rate of 0.8. It indicates that the dropout layers help in reducing the overfitting influence. However, if we use lower keep rates or increase the layers using dropout layer, the accuracy declines. The reason is that the features extracted by the no-dropout-layer structure are redundant. For the network without dropout layer, we directly use it to classify the nodules, and the overfitting problem causes the generalization to be reduced. For the networks which dropout more neurons, the remaining information would be incomplete; especially for the keep rate of 0.5, where only half of neurons are kept, both the details and semantic information are lacking. In this way, incomplete features lead to lower classification accuracies. We usually use the dropout rate of 0.8; however, its choice is vague for different tasks and it can be adjusted if necessary. For this task, we adopted the general number of 0.8 and achieved the best performance.

#### 4.1.2. Evaluation of the ML-xResNet

We evaluated the stability and efficiency of the proposed ML-xResNet by using six-fold cross-validation. Then, we show the classification results for each class and show the extracted features of each level. Then, we evaluate the performance of the proposed ML-xResNet with state-of-the-art methods.

The validation of the reliability and robustness of ML-xResNet is by the six-fold cross-validation since the training to testing ratio is 85:15. We split the dataset into six groups of 15% data, which were used as the testing set one after another, with the rest was the training set. Finally, the average classification accuracy is 85.88%, achieving state-of-the-art performance. Then, we evaluated the stability of the proposed network based on the results of the six-fold cross validation by calculating the standard deviation. We obtained 0.8588±0.0121. Therefore, our proposed method is stable in this task, and we take the best-performed result as our final result. Besides, we use fewer layers than the DenseNet and its variations but achieve the best performance. The three-level cross DenseNet takes many concatenation operations, and the number of features grows after each epoch, which slows down the computation speed. We show better efficiency than the three-level cross DesNet in both compute speed and classification performance.

Moreover, we also tested the classification accuracy for each class; the results are shown in Table 4. It reveals that the proposed ML-xResNet has the best performance for classifying malignant nodules and reaches 86.92%, which is 1.04% higher than the average accuracy of the three classes. For benign nodules, the classification accuracy is close to the average accuracy of 85.88%. However, for the indeterminate nodules, the accuracy is 0.87% lower than it. The reason is that, compared to the indeterminate nodules, the benign and malignant nodules have more distinguishable features. For some of the indeterminate nodules, even the radiologists who diagnosed the nodules gave the opposite annotations. It means that those nodules have both benign and malignant characteristics, and they are hard to confirm by the CT images and need further diagnosis. Therefore, the proposed ML-xRes strategy is efficient to identify them using the fusing features through both the details and the semantic information.

Finally, we compared the performance of the proposed ML-xResNet with state-of-the-art algorithms. The MC-CNN adopts the multi-crop technique in the first pooling layer to extract multi-scale features of the input. It consists of three convolution and pooling layers, and each layer extracts 64 features. We also compared with the adjusted DenseNet [35], which performed very well in the classification task, as well as with its three-level version and three-level cross-version.

The comparison testing results are tabulated in Table 5. It is shown that the adjusted DenseNet only achieves an accuracy of 68.90%. When we applied the multi-level and the multi-level cross-connection strategies, the performance is improved obviously. Based on the results of the DenseNet and its variants, we verified that the multi-level structure is better than the single level one in our task, and the multi-level cross strategy performs better than the multi-level only. Moreover, the accuracy of our proposed ML-xResNet approach is 2.19% higher than the three-level cross DenseNet. Besides, the three-level cross DenseNet takes more time for training and testing processes. MC-CNN performs worst than the single level DenseNet, 23.42% lower than the ML-xResNet. It indicates that our proposed ML-xRes strategy is more powerful at extracting distinguished features of the nodules. Therefore, our proposed ML-xResNet achieves the best performance for ternary classification.

### 4.2. Binary Classification

In this section, we extend our work to binary classification. The nodules were classified into two classes: binary and malignant. Besides, the network we used in this task is entirely the same as the one in ternary classification, the same structure and the same hyperparameters. It also has the best performance in the binary task. We evaluated the performance of ML-xResNet in the binary classification with the state-of-the-art models using four assessment criteria: accuracy, sensitivity, specificity, and AUC.

We compared with the mentioned MC-CNN and other state-of-the-art networks. da Nóbrega et al. [18] employed multiple of pre-trained models to extract features of the lung nodules, which are then classified by six kinds of classifiers. Finally, the combination of ResNet50 and SVM-RBF achieves the best performance. The MoDenseNet consists of two pathways 3D DenseNet; different scales of inputs are inputted into two networks. Hussein et al. [22] proposed a novel supervised and unsupervised method for the lung nodule classification. They obtained the best result by using the supervised structure, which utilizes a multi-task algorithm for both classification and attributes score regression. The deep local–global network consists of two combination blocks of the ResNet block and the Non-local block, followed by a global average pooling layer to summarize the features. Finally, a sigmoid classifier is used to get the result. The multi-view CNN trains three CNNs for the three views of the input, respectively, and then, uses an OR operation to get the final classification result.

The comparison result is shown in Table 6. It shows that our proposed ML-xResNet get the best performance in accuracy, sensitivity, and AUC. MC-CNN performs best in the specificity. In the area of disease diagnosis, sensitivity presents the percentage of sick people who are correctly identified as having the condition, while the specificity presents the percentage of healthy people who are correctly identified as not having the condition. Therefore, for the CAD, an assistive tool for diagnosing, it is more important to classify the images with diseases as correctly as possible. For the state-of-the-art methods, only MoDenseNet achieves an accuracy above 90%. Besides, the transfer learning and multi-task learning combination method in [22] shows the worst performance among all the structures. Apart from the Deep local–global network, the rest of the six algorithms can be roughly divided into three categories. ResNet50 with SVM-RBF and the method in [22] both use the transfer learning method to classify the nodules. MoDenseNet and the Multi-view CNN are mainly based on the multi-view input strategy to improve the effectiveness of the features. MC-CNN and our proposed ML-xResNet mainly consider extracting multi-scale features of the input. The Deep local–global network uses a combination of ResNet and non-local blocks to classify the nodules. Compared to other kinds of methods, our proposed strategy shows better generalization ability. By using multi-size filters to extract features, the nodules with various sizes have more appropriate receptive fields, which improves the classifier’s distinguishing ability. Another reason the generalization ability is better is that we use more data than the other methods. Most of the methods only use hundreds of data, and at most thousands. Small datasets cause the network to only fit a few kinds of data and have low generalization. Therefore, the proposed ML-xResNet shows the best performance for binary classification.

Above all, according to all the experiment results, it can be proven that our proposed model has the best performance in both the ternary and the binary classification tasks.

## 5. Discussion

Usually, the objects we study are with various sizes and morphologies, especially the organs, tissue, and lesions. Researchers have taken some measures to tackle this problem, such as the multi-view networks and the multi-scale networks. However, mostly, it is difficult or costly to get multi-views or scales of an object of medical image. Therefore, we turn our idea to extract multi-scale features of a fixed view image instead of the fixed scale features of multi-view/multi-scale inputs. We propose to achieve it by the ML-xResNet algorithm, which extracts multi-scale features for different sizes and morphologies without any changing for the inputs.

Based on the experiment results, we found that the proposed ML-xResNet accomplishes the best performance in both ternary and binary classification tasks using the same architecture and hyperparameters. Similarly, Shen et al. [17] also implemented their method, MC-CNN, in both the above tasks; however, their performance of binary classification was 24.86% higher than of ternary classification, while this gap is only 7.1% using our model. It reveals that our method has better performance for similar tasks. It indicates that our multi-level xRes strategy can explore and extract more powerful features than their multi-crop pooling strategy; therefore, our method has a better representation power for the lung nodule images.

According to the results in Table 1, we know that the multi-level structure is effective for solving the lung nodule classification problem. However, with the growth of the levels, the parameters are too many to get better performance. The overfitting problem shows more influence on the result than the improvement with more levels and scales of features. This situation also appears in Table 2. Then, we use the dropout technique in our model. However, as shown, when we use dropout layers after all the convolution layers, it shows a worse result than only adding them below the odd layers. The above indicates that, if the network is too wide or too deep, the performance would be worse with the growth of the parameters. The reason is that, if the network is too wide, it will produce redundant information, which causes the overfitting problem; if the network is too deep, the details of the features would be only few pixels or even disappear, which leads to the features not being distinguished, as well as to the overfitting problem. To avoid the too wide or too deep architecture requires more experience and experiments for different tasks, which is what we did in this study. Moreover, the number of dropout layers or dropout rate also reflects the performance of the network. Therefore, selecting appropriate hyperparameters is very important.

After the evaluation through the experiments, it is indicated that the enhancement of performance attributes to the proposed multi-scale convolution method, which helps the network extract more effective features, is also attributed to the strategy of cross residuals, which guarantees all scales of features share information during training to improve the identification ability. Besides, the selection of dropout parameter helps the network to minimize the influence of overfitting. Furthermore, the proposed method is referential to the medical imaging tasks whose classification objects have a variety of sizes and morphologies, and our experimental procedure also displays the exploration process.

## 6. Conclusions

In this paper, we introduce a novel deep learning computational architecture, multi-level cross ResNet, to classify the malignancy of lung nodules. We studied both ternary classification, which includes benign, indeterminate, and malignant categories, and binary classification, with benign and malignant classes. The evaluation results show that the multi-level and cross residual structures help to extract multi-scale features and fuse them to improve the performance in both binary and ternary classifications. In the future, we will consider combining some unsupervised deep features on patch level to enrich the features, as it have been proven that multi-task learning can improve performance. We will also study how to classify the nodules directly using the full size of images. Since nodules are small compared with a lung, to improve the classification performance, we would adopt the attention mechanism or use a segmentation method to highlight the nodules in the full size of the image.

## Figures and Tables

**Table 1:** Comparison of different levels and convolutional kernel sizes xResNet in accuracy, the best result is in bold.

Levels	Convolutional Kernel Size	Accuracy (%)
Single Level	3/7/11	79.75/83.48/84.60
Two Levels	3_7/3_11/7_11	84.10/84.90/84.46
Three Levels	3_7_11	**85.88**
Four Levels	3_5_7_11	84.83

**Table 2:** Comparison of different numbers of ML-xRes blocks of ML-xResNet in accuracy, the best result is in bold.

Number of xRes Blocks	Number of Features of Each Layer	Accuracy (%)
1	64, 64, 128	81.77
2	64, 64, 128, 128, 256	**85.88**
3	64, 64, 128, 128, 256, 256, 512	85.06
4	64, 64, 128, 128, 256, 256, 512, 512, 512	83.25

**Table 3:** Comparison of different dropout keep rates and dropout layers of ML-xResNets in accuracy, the best result is in bold.

Number Dropout Layers	Dropout Keep Rates	Accuracy (%)
0	1.0	84.52
3	0.8	**85.88**
3	0.6	84.14
3	0.5	83.21
5	0.8	85.33

**Table 4:** Classification accuracy for each class, the best result is in bold.

Malignancy	Accuracy (%)
benign	85.86
indeterminate	85.01
malignant	**86.92**

**Table 5:** Compared with the state-of-the-art approaches in accuracy, the best result is in bold.

Models	Accuracy (%)
DenseNet [35]	68.90
Three-level DenseNet	79.67
Three-level cross DenseNet	83.69
MC-CNN [17]	62.46
this work	**85.88**

**Table 6:** Compared with the state-of-the-art methods for binary classification, the best results are in bold.

Models	Accuracy (%)	Sensitivity (%)	Specificity (%)	AUC(%)
MC-CNN [17]	87.14	77.00	**93.00**	93.00
ResNet50 + SVM-RBF [18]	88.41	85.38	–	93.19
MoDenseNet [19]	90.40	90.47	–	95.48
Deep local–global Network [20]	88.46	88.66	–	95.62
Multi-view CNN [21]	89.90	85.26	90.66	94.85
Paper [22]	81.73	78.24	85.97	–
This work	**92.19**	**92.10**	91.50	**97.05**

## References

[1] Ferlay J., Ervik M., Lam F., Colombet M., Mery L., Pi neros M., Znaor A., Soerjomataram I., Bray F. (2018). Global Cancer Observatory: Cancer Today.

[2] Smith R.A., Andrews K.S., Brooks D., Fedewa S.A., Wender R.C. (2019). Cancer screening in the United States, 2019: A review of current American Cancer Society guidelines and current issues in cancer screening. CA Cancer J. Clin..

[3] Bach P.B., Silvestri G.A., Hanger M., Jett J.R. (2007). Screening for lung cancer: ACCP evidence-based clinical practice guidelines. Chest.

[4] Larici A.R., Farchione A., Franchi P., Ciliberto M., Cicchetti G., Calandriello L., del Ciello A., Bonomo L. (2017). Lung nodules: Size still matters. Eur. Respir. Rev..

[5] Hansell D.M., Bankier A.A., MacMahon H., McLoud T.C., Muller N.L., Remy J. (2008). Fleischner Society: Glossary of terms for thoracic imaging. Radiology.

[6] Greenspan H., Van Ginneken B., Summers R.M. (2016). Guest editorial deep learning in medical imaging: Overview and future promise of an exciting new technique. IEEE Trans. Med. Imaging.

[7] Bakator M., Radosav D. (2018). Deep learning and medical diagnosis: A review of literature. Multimodal Technol. Interact..

[8] Sahiner B., Pezeshk A., Hadjiiski L.M., Wang X., Drukker K., Cha K.H., Summers R.M., Giger M.L. (2019). Deep learning in medical imaging and radiation therapy. Med. Phys..

[9] Litjens G., Kooi T., Bejnordi B.E., Setio A.A.A., Ciompi F., Ghafoorian M., Van Der Laak J.A., Van Ginneken B., Sánchez C.I. (2017). A survey on deep learning in medical image analysis. Med. Image Anal..

[10] Schlemper J., Oktay O., Schaap M., Heinrich M., Kainz B., Glocker B., Rueckert D. (2019). Attention gated networks: Learning to leverage salient regions in medical images. Med. Image Anal..

[11] Minaee S., Abdolrashidi A. (2019). Deep-emotion: Facial expression recognition using attentional convolutional network. arXiv.

[12] Iqbal T., Ali H. (2018). Generative adversarial network for medical images (MI-GAN). J. Med. Syst..

[13] Minaee S., Wang Y., Choromanska A., Chung S., Wang X., Fieremans E., Flanagan S., Rath J., Lui Y.W. A deep unsupervised learning approach toward MTBI identification using diffusion MRI. Proceedings of the 40th Annual International Conference of the IEEE Engineering in Medicine and Biology Society (EMBC).

[14] Halder A., Dey D., Sadhu A.K. (2020). Lung Nodule Detection from Feature Engineering to Deep Learning in Thoracic CT Images: A Comprehensive Review. J. Digit. Imaging.

[15] Minaee S., Wang Y., Aygar A., Chung S., Wang X., Lui Y.W., Fieremans E., Flanagan S., Rath J. (2019). MTBI identification from diffusion MR images using bag of adversarial visual features. IEEE Trans. Med. Imaging.

[16] Shen W., Zhou M., Yang F., Yang C., Tian J. (2015). Multi-scale convolutional neural networks for lung nodule classification. Proceedings of the International Conference on Information Processing in Medical Imaging.

[17] Shen W., Zhou M., Yang F., Yu D., Dong D., Yang C., Zang Y., Tian J. (2017). Multi-crop convolutional neural networks for lung nodule malignancy suspiciousness classification. Pattern Recognit..

[18] da Nóbrega R.V.M., Peixoto S.A., da Silva S.P.P., Rebouças Filho P.P. Lung nodule classification via deep transfer learning in CT lung images. Proceedings of the IEEE 31st International Symposium on Computer-Based Medical Systems (CBMS).

[19] Dey R., Lu Z., Hong Y. Diagnostic classification of lung nodules using 3D neural networks. Proceedings of the IEEE 15th International Symposium on Biomedical Imaging (ISBI 2018).

[20] Al-Shabi M., Lan B.L., Chan W.Y., Ng K.H., Tan M. (2019). Lung nodule classification using deep Local—Global networks. Int. J. Comp. Assist. Radiol. Surg..

[21] El-Regaily S.A., Salem M.A.M., Aziz M.H.A., Roushdy M.I. (2019). Multi-view Convolutional Neural Network for lung nodule false positive reduction. Expert Syst. Appl..

[22] Hussein S., Kandel P., Bolan C.W., Wallace M.B., Bagci U. (2019). Lung and pancreatic tumor characterization in the deep learning era: Novel supervised and unsupervised learning approaches. IEEE Trans. Med. Imaging.

[23] Liu L., Dou Q., Chen H., Qin J., Heng P.A. (2019). Multi-Task Deep Model with Margin Ranking Loss for Lung Nodule Analysis. IEEE Trans. Med. Imaging.

[24] Lyu J., Ling S.H. Using multi-level convolutional neural network for classification of lung nodules on CT images. Proceedings of the 40th Annual International Conference of the IEEE Engineering in Medicine and Biology Society (EMBC).

[25] Szegedy C., Liu W., Jia Y., Sermanet P., Reed S., Anguelov D., Erhan D., Vanhoucke V., Rabinovich A. Going deeper with convolutions. Proceedings of the IEEE Conference on Computer Vision and Pattern Recognition.

[26] He K., Zhang X., Ren S., Sun J. Deep residual learning for image recognition. Proceedings of the IEEE Conference on Computer Vision and Pattern Recognition.

[27] Armato S.G., McLennan G., Bidaut L., McNitt-Gray M.F., Meyer C.R., Reeves A.P., Zhao B., Aberle D.R., Henschke C.I., Hoffman E.A. (2011). The lung image database consortium (LIDC) and image database resource initiative (IDRI): A completed reference database of lung nodules on CT scans. Med. Phys..

[28] Lin T.Y., Dollár P., Girshick R., He K., Hariharan B., Belongie S. Feature pyramid networks for object detection. Proceedings of the IEEE Conference on Computer Vision and Pattern Recognition.

[29] Newell A., Yang K., Deng J. (2016). Stacked hourglass networks for human pose estimation. Proceedings of the European Conference on Computer Vision.

[30] Clark K., Vendt B., Smith K., Freymann J., Kirby J., Koppel P., Moore S., Phillips S., Maffitt D., Pringle M. (2013). The Cancer Imaging Archive (TCIA): Maintaining and operating a public information repository. J. Digit. Imaging.

[31] Reeves A.P., Biancardi A.M. The Lung Image Database Consortium (Lidc) Nodule Size Report. http://www.via.cornell.edu/lidc/.

[32] Abadi M., Barham P., Chen J., Chen Z., Davis A., Dean J., Devin M., Ghemawat S., Irving G., Isard M. Tensorflow: A system for large-scale machine learning. Proceedings of the 12th USENIX Symposium on Operating Systems Design and Implementation (OSDI 16).

[33] Dong H., Supratak A., Mai L., Liu F., Oehmichen A., Yu S., Guo Y. Tensorlayer: A versatile library for efficient deep learning development. Proceedings of the 25th ACM international conference on Multimedia.

[34] Rubinstein R., Kroese D. (2004). The Cross-Entropy method: A Unified Approach to Combinatorial Optimization, Monte-Carlo Simulation, and Machine-Learning.

[35] Huang G., Liu Z., Van Der Maaten L., Weinberger K.Q. Densely connected convolutional networks. Proceedings of the IEEE Conference on Computer Vision and Pattern Recognition.

